# Schisandrin A from Schisandra chinensis Attenuates Ferroptosis and NLRP3 Inflammasome-Mediated Pyroptosis in Diabetic Nephropathy through Mitochondrial Damage by AdipoR1 Ubiquitination

**DOI:** 10.1155/2022/5411462

**Published:** 2022-08-11

**Authors:** Xiaohu Wang, Qin Li, Bangzhi Sui, Maodi Xu, Zhichen Pu, Teng Qiu

**Affiliations:** ^1^Drug Clinical Evaluation, First Affiliated Hospital of Wannan Medical College, Wuhu, Anhui 241001, China; ^2^Department of Endocrinology, First Affiliated Hospital of Wannan Medical College, Wuhu, Anhui 241001, China; ^3^Key Laboratory of Non-coding RNA Transformation Research of Anhui Higher Education Institution, Wannan Medical College, Wuhu 241001, China; ^4^Department of Pediatric Surgery, First Affiliated Hospital of Wannan Medical College, Wuhu, Anhui 241001, China; ^5^Department of Urology Surgery, First Affiliated Hospital of Wannan Medical College, Wuhu, Anhui 241001, China

## Abstract

*Schisandra chinensis*, as a Chinese functional food, is rich in unsaturated fatty acids, minerals, vitamins, and proteins. Hence, this study was intended to elucidate the effects and biological mechanism of Schisandrin A from *Schisandra chinensis* in DN. C57BL/6 mice were fed with a high-fat diet and then injected with streptozotocin (STZ). Human renal glomerular endothelial cells were stimulated with 20 mmol/L d-glucose for DN model. Schisandrin A presented acute kidney injury in mice of DN. Schisandrin A reduced oxidative stress and inflammation in model of DN. Schisandrin A reduced high glucose-induced ferroptosis and reactive oxygen species (ROS-)-mediated pyroptosis by mitochondrial damage in model of DN. Schisandrin A directly targeted AdipoR1 protein and reduced LPS+ATP-induced AdipoR1 ubiquitination in vitro model. Schisandrin A activated AdipoR1/AMPK signaling pathway and suppressed TXNIP/NLRP3 signaling pathway in vivo and in vitro model of DN. Conclusively, our study revealed that Schisandrin A from *Schisandra chinensis* attenuates ferroptosis and NLRP3 inflammasome-mediated pyroptosis in DN by AdipoR1/AMPK-ROS/mitochondrial damage. Schisandrin A is a possible therapeutic option for DN or other diabetes.

## 1. Introduction

Type 2 diabetes is a common syndrome of metabolic and endocrine dysfunction, with major pathological changes of insulin resistance and *β* cell dysfunction [1]. With the rapidly increasing incidence worldwide, it is estimated that the number of diabetic patients will reach 300 million by 2025 [2]. Among them, about one-third of diabetes later developed into diabetic nephropathy, which is the main cause of end-stage renal disease (ESRD) [3]. At present, there is no clear etiology and pathogenesis of DN, which leads to limitations in the clinical treatment of DN [3].

DN is a chronic inflammatory disease characterized by inflammatory cell infiltration and overexpression of proinflammatory factors [4]. When chronic inflammation occurs in tissues or organs, macrophages will accumulate in large numbers [5]. Inflammatory infiltration of macrophages in glomeruli and tubules is the main pathological feature of diabetic kidney injury [6, 7].

The method of cell death, including apoptosis, autophagy, iron death, necrosis, and scorch, provides new directions and targets for DN therapy, which has become a research hotspot in this field [8]. Among them, iron death is a process of programmed cell death that is different from apoptosis and autophagy, characterized by abnormally increased intracellular lipid oxygen free radicals [9, 10].

Gasdermin D (GSDMD) protein is the common substrate of all inflammatory caspases, which can independently mediate the release of interleukin-1 beta (IL-1*β*) and other inflammatory mediators [11]. Inflammatory caspases cleave GSDMD/E at specific sites [12]. The effect mechanism of pyrolysis cell membrane rupture and cell disintegration is to activate the punching activity [12]. The immune inflammatory disease DN is mediated by a variety of inflammatory factors [13]. As an important factor of chronic kidney injury, inflammation plays an important mediating role in the whole process of pyrolysis and DN [13].

*Schisandra chinensis*, as a Chinese functional food, is rich in unsaturated fatty acids, minerals, vitamins, and proteins, and used as a tonic and sedative agent in traditional Chinese medicine [14–16]. Schisandrin A is one of the lignans isolated from the dried fruits of *Schisandra chinensis* [17, 18]. It has a wide range of pharmacological effects in antioxidation, inhibition of apoptosis, and regulation of immunity [19, 20]. Hence, this study was intended to elucidate the effects and biological mechanism of Schisandrin A from *Schisandra chinensis* in DN.

## 2. Materials and methods

### 2.1. Animal Care Model

Animals were approved by the Animal Care and Use Committee of Yijishan Hospital of Wannan Medical College (No. LLSC-2021-112). All C57BL/6 mice (male, 5-6 weeks, 18-20 g) were obtained from and animal testing center of Qinglongshan (Nanjing, Suzhou, China). C57BL/6 mice were randomly assigned to five groups: sham group (*n* = 8), model group (*n* = 8), and 25/50/100 mg/kg Schisandrin A treatment group (*n* = 8/group). All mice of sham group were fed with normal diet. All mice of model group and 5/50/100 mg/kg Schisandrin A treatment group were fed with a high-fat diet (HFD) for 12 weeks and then injected with STZ (30 mg/kg of streptozotocin; Sigma-Aldrich, St. Louis, MO, USA) i.p. for 7 consecutive days. Blood glucose levels of mice were measured using 16.7 mmol/l after one week of the final injection. Blood glucose levels of mice were measured using 16.7 mmol/l after one week of the final injection. Then, all mice of Schisandrin A group were administration with 25/50/100 mg/kg Schisandrin A for 8 weeks. Mice were killed under anesthesia, and their kidneys are taken for analysis.

### 2.2. Histological Examination

Kidney tissue samples were fixed in 4% paraformaldehyde, paraffin-embedded, and then sectioned into 5 *μ*M slices for Masson staining, PAS staining, or TUNEL staining. Kidney tissue samples were observed using fluorescence microscope (Zeiss Axio Observer A1, Germany).

Kidney slices were used for collagen IV by immunohistochemistry. Kidney slices are incubated with primary antibodies against collagen IV (CST, USA) at 1 : 200 dilution overnight at 4°C with the appropriate secondary antibodies (1 : 200 Santa Cruz, CA, USA). Kidney tissue samples were observed using fluorescence microscope (Zeiss Axio Observer A1, Germany).

### 2.3. Kidneys Function

Urine albumin and creatinine were measured on a spot urine sample by ELISA kit (Nanjing Jiancheng Bioengineering Research Institute). Kidney function was measured using a Vevo 770 high-resolution imaging system (Visual Sonics, Canada) equipped with a high-frequency ultrasound probe (RMV-707B).

### 2.4. Lentivirus Injection

The lentiviral vectors carrying TXNIP plasmid for TXNIP or a short hairpin RNA (shRNA) for AdipoR1 were designed and chemically synthesized by Hanyin Biotechnology Limited Company (Shanghai, China). The constructs were diluted to a total volume of 200 *μ*L (5 × 107 TU) and administered into the mice through tail vein injection.

### 2.5. Cell Culture and Treatment

Human renal glomerular endothelial cells (HRGECs) were seeded in culture dish with RPMI 1640 (Gibco) supplemented with 10% FBS (Gibco) under a humidified 5% (v/v) CO2 atmosphere at 37°C. HRGECs are stimulated with 20 mmol/L d-glucose for DN model. Next, HRGECs are stimulated with 20 mmol/L d-glucose and 25/50/100 *μ*M of Schisandrin A.

The transfections were performed using Lipofectamine 2000 (Thermo Fisher Scientific). After 48 h of transfection, HRGECs are stimulated with 20 mmol/L d-glucose or 25/50/100 *μ*M of Schisandrin A.

### 2.6. ELISA Assay

Blood, tissue, or cell samples were collected and used to measure IL-1*α*, INF-*γ*, TNF-*α*, IL-6, and IL-1*β* levels using IL-1*α*, INF-*γ*, TNF-*α*, IL-6, and IL-1*β* ELISA kits (Nanjing Jiancheng Biological Engineering Institute, Nanjing, China) following the manufacturer's instructions. ROS production was evaluated by measuring ROS levels kits (S0033S, Beyotime Biotechnology) following the manufacturer's instructions. Calcein AM/CoCl2 assay and JC-1 disaggregation were evaluated by mitochondrial permeability transition pore assay kit (C2009S, Beyotime Biotechnology) and enhanced mitochondrial membrane potential assay kit with JC-1 (C2003S, Beyotime Biotechnology).

### 2.7. Flow Cytometry for Apoptosis

Cells were resuspended with 100 *μ*L binding buffer and stained with propidium iodide (556547, BD Pharmingen, San Diego, USA) for 15 min at room temperature. Cells were collected and analyzed by flow cytometer (BD Biosciences, San Jose, USA).

### 2.8. Cell Counting Kit-8 (CCK8) Assay and LDH Activity

HRGECs with different polysaccharides were cultured in a 96-well plate for corresponding time points and then incubated with CCK-8 reagent or LDH activity kit. The proliferation or LDH activity levels were assessed via the absorbance using a microplate reader (Thermo Fisher Scientific) at 450 nm.

### 2.9. PI Staining and Calcein/PI Staining

Cells were washed with PBS and fixed with 4% paraformaldehyde. Cells were incubated with PI staining (ST512, Beyotime Biotechnology) or calcein/PI staining (C2015S, Beyotime Biotechnology). Cell samples were observed using fluorescence microscope (Zeiss Axio Observer A1, Germany).

### 2.10. Quantitative Polymerase Chain Reaction (qPCR)

Total RNAs were isolated with RNA isolator total RNA extraction reagent (Takara), and cDNA was synthesized using PrimeScipt RT Master Mix (Takara). qPCR were performed with the ABI Prism 7500 sequence detection system according to the Prime-ScriptTM RT detection kit. PCR primers: E-cadherin, 5′-TACAACGACCCAACCCAA-3′ (sense) and 5′-TCCTCCGAAGAAACAG CA-3′ (antisense); periostin, 5′-CTGCCAAACAAGTTATTGAGCTGGC-3′ (sense) and 5′-AATAATGTCCAGTCTCCAGGTTG-3′ (antisense); GAPDH, 5′-AGAAGGCTGGGGCTCATTTG-3′ (sense) and 5′-AGGGGCCATCCACA GTCTTC-3′ (antisense). Relative levels of the sample mRNA expression were calculated and expressed as 2-DDCt.

### 2.11. Western Blotting Analysis

Tissue samples or cell samples or supernatant samples were splitted using RIPA assay (Beyotime) in ice. Total proteins were quantified using BCA assay (Beyotime) and were electrophoresed on 10% SDS-acrylamide gels. Total proteins were transferred to nitrocellulose membranes, and membranes were blocked with 5% nonfat milk in TBS for 1 h at 37°C. Membranes were incubated with AdipoR1 (ab50675, 1 : 2000, abcam), AMPK (ab32047, 1 : 2000, abcam), p-AMPK (ab133448, 1 : 1000, abcam), TXNIP (ab188865, 1 : 2000, abcam), NRF2 (ab62352, 1 : 2000, abcam), HO-1 (ab52947, 1 : 2000, abcam), sOD2 (ab68155, 1 : 2000, abcam), GPX4 (ab41787, 1 : 2000, abcam), GSDMD (ab209845, 1 : 2000, abcam), NLRP3 (sc-66846, 1 : 500, Santa Cruz, USA), caspase-1 (sc-1780, 1 : 500, Santa Cruz, USA), IL-1*β* (sc-12742, 1 : 500, Santa Cruz, USA), and *β*-actin (BS6007MH, 1 : 5000, Bioworld Technology, Inc.) at 4°C overnight. The membranes were incubated with horseradish peroxidase-conjugated secondary antibodies (sc-2004 or sc-2005, 1 : 5000, Santa Cruz, USA) for 1 h at 37°C after washing with TBST for 15 min. Protein was measured using an enhanced chemiluminescence system (ECL, Beyotime) and analyzed using an Image Lab 3.0 (Bio-Rad Laboratories, Inc.).

### 2.12. Electron Microscope

Cells were fixed in 0.2 M phosphate buffer (KH2PO4/Na2HPO4, pH 7.5) and supplemented with 2.5% glutaraldehyde (G5882, Sigma-Aldrich) for 1 h at room temperature. Cells were post-fixed for 60 min in 1% osmium tetroxide (75632, Sigma-Aldrich) and dehydrated in gradient ethanol solutions (50-100%) for 15 min. Cells were infiltrated sequentially in ethanol: spurr-resin (Polyscience, Warrington, USA) (1 : 1) for 30 min and then infiltrated sequentially in ethanol: spurr-resin for 30 min for 30 min, then 100% spurr-resin for 3-4 h, and finally 100% spurr-resin for 24 h at 60°C. Ultrathin sections were isolated on nickel grids and stained for 10 min in 2% uranyl acetate and then in Reynolds lead citrate for 15 min. Ultrathin sections were observed using a Hitachi H7650 transmission electron microscope (Tokyo, Japan).

### 2.13. Microscale Thermophoresis (MST), Thermal Shift Assay (TSA), and Cellular Thermal Shift Assay (CETSA)

MST, TSA, and CETSA were executed as described previously [21].

*0.10* mg*/mL WT AdipoR1 protein was used with or without 0.30* mmol*/L Schisandrin A in PBS. Data were analyzed with the differential scanning fluorimetry analysis tool (Microsoft Excel-based) by using the curve-fitting software XLfit 5 (*http://www.idbs.com*, ID Business Solutions Ltd.).* Mut AdipoR1, indicating SER-205, ARG-267, LYS-206, TRP-255, and TYR-209 *plasmids*, *were transfected to HEK 293*T *cells with LTX reagent and PLUS reagent* (Invitrogen). After 8 h, the cells were cultured with 100 *μ*mol/L Schisandrin A for 2 h, and control cells were incubated with the same volume of PBS. Cell samples were cultivated in PBS (containing 1 mmol/L PMSF) and heated with a thermal gradient from 37 to 67°C for 3 min. 20 *μ*L supernatant was used to Western blotting. CETSA and the thermal stability were performed using GraphPad Prism software (GraphPad Diego, CA, USA).

### 2.14. Statistical Analysis

Data were expressed as mean ± SEM. Data analysis were performed by Student's t-test or one-way ANOVA followed by Tukey's post-test using GraphPad Prism 8. *P* values <0.05 were considered statistically significant.

## 3. Results

### 3.1. Schisandrin A Presented STZ-Induced DN

**Figure 1:**
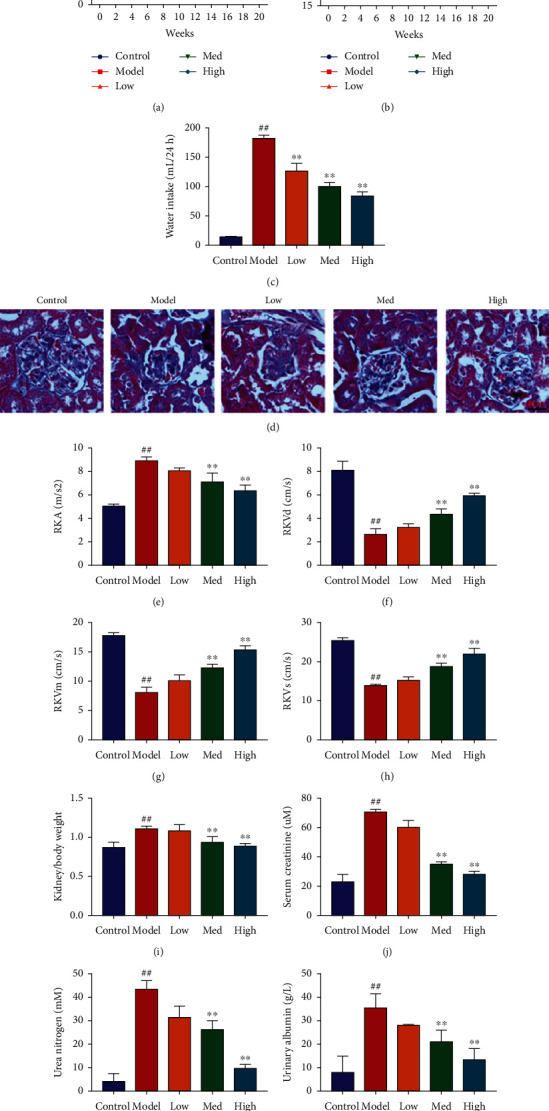
Schisandrin A presented STZ-induced DN. (a) blood glucose; (b) body weight; (c) water intake (24 h); (d) glomerulus injury (Masson staining); (e–h) RKA, RKVd, RKVm, and RKVs levels; (i) kidney/body weight; (j) serum creatinine; (k) urea nitrogen; and (l) urinary albumin levels in STZ-induced DN. Control, sham control mice group; model, STZ-induced mice DN group; low, mice DN by treatment with 25 mg/kg of Schisandrin A group; med, mice DN by treatment with 50 mg/kg of Schisandrin A group; high, mice DN by treatment with 100 mg/kg of Schisandrin A group; ##*P* < 0.01 versus control group; ∗∗*P* < 0.01 versus STZ-induced mice DN group.

The study explored the effects of Schisandrin A in STZ-induced DN. These Schisandrin A could reduce blood glucose, increased body weight, inhibited water intake (24 h), and presented glomerulus injury in STZ-induced DN through dose-dependent manner (Figures 1(a)–1(d)). Then, these Schisandrin A could reduce RKA level and increased RKVd, RKVm, and RKVs levels in STZ-induced DN through dose-dependent manner (Figures 1(e)–1(h)). Meanwhile, these Schisandrin A reduced kidney/body weight and serum creatinine and inhibited urea nitrogen and urinary albumin levels in STZ-induced DN through dose-dependent manner (Figures 1(i)–1(l)). Thus, these results showed that Schisandrin A from *Schisandra chinensis* might present STZ-induced DN.

### 3.2. Schisandrin A Reduced Acute Kidney Injury in Mice of DN

**Figure 2:**
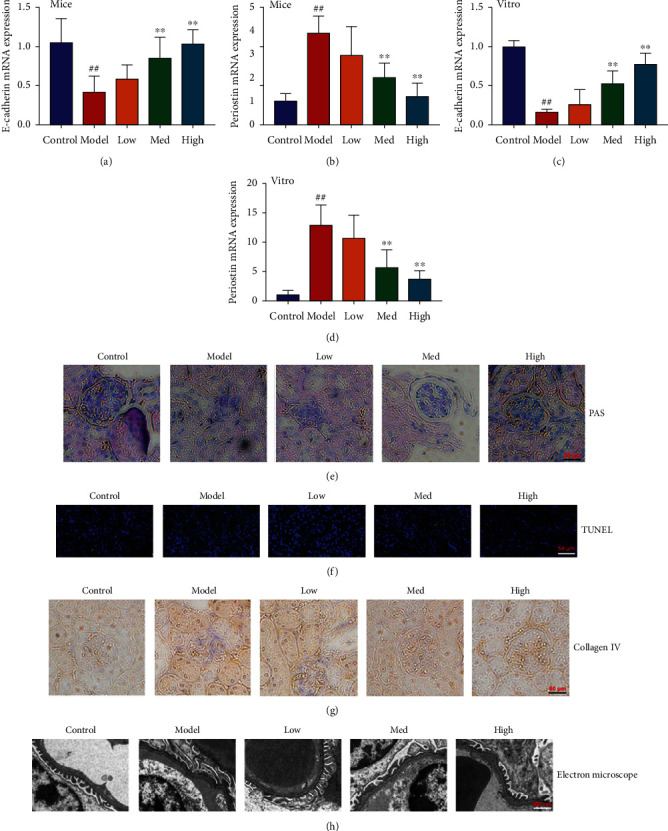
Schisandrin A reduced acute kidney injury in mice of DN. (a) E-cadherin mRNA expression; (b) periostin mRNA expression in mice of DN; (c) E-cadherin mRNA expression, (d) periostin mRNA expression in vitro model of DN; (e) PAS staining; (f) TUNEL staining and (g) collagen IV staining in mice of DN; and (h) podocyte ultrastructure changes using electron microscope in mice of DN. Control, sham control mice group; model, STZ-induced mice DN group; low, mice DN by treatment with 25 mg/kg of Schisandrin A group; med, mice DN by treatment with 50 mg/kg of Schisandrin A group; high, mice DN by treatment with 100 mg/kg of Schisandrin A group; ##*P* < 0.01 versus control group; ∗∗*P* < 0.01 versus STZ-induced mice DN group.

Next, we further explored that the effects of Schisandrin A regulated acute kidney injury in mice of DN. In mice model of DN or in vitro model of DN, these Schisandrin A reduced E-cadherin mRNA expression and increased periostin mRNA expression (Figures 2(a)–2(d)). PAS, TUNEL, and collagen IV stainings showed that these Schisandrin A inhibited glomerulus injury and renal cell apoptosis in mice model of DN (Figures 2(e)–2(g)). Electron microscope showed that Schisandrin A inhibited Masson-positive area and effacement of the podocyte foot processes in mice model of DN (Figure 2(h)). Taken together, these results suggest that Schisandrin A reduced acute kidney injury in mice model of DN.

### 3.3. Schisandrin A reduced oxidative stress and inflammation in model of DN

**Figure 3:**
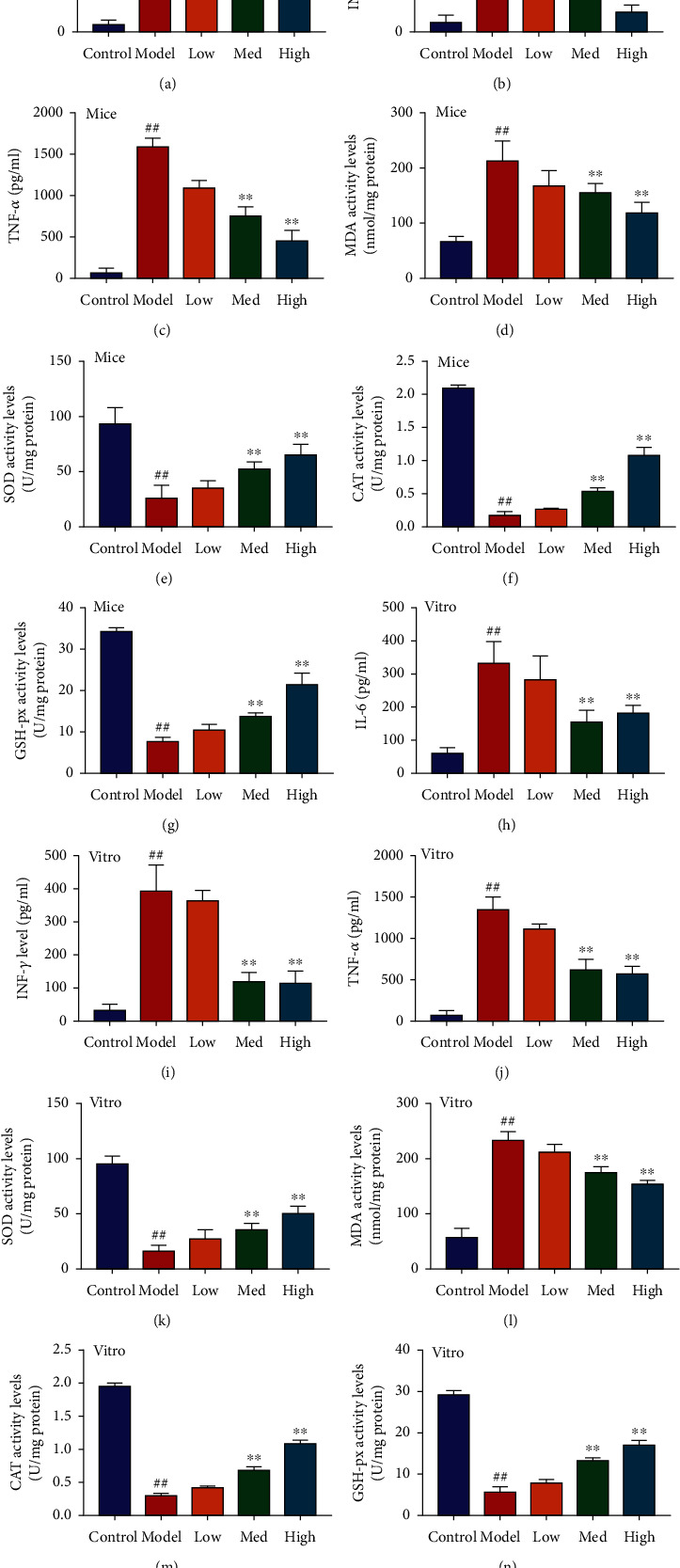
Schisandrin A reduced oxidative stress and inflammation in model of DN. (a, b, and c) IL-6, INF-*γ*, and TNF-*α*; (d, e, f, and g) MDA, SOD, CAT, and GHS levels in mice of DN; (h, i, and j) IL-6, INF-*γ*, and TNF-*α*; and (k, l, m, and n) MDA, SOD, CAT, and GHS levels in vitro model of DN. Control, sham control mice group; model, STZ-induced mice DN group; low, mice DN by treatment with 25 mg/kg of Schisandrin A group; med, mice DN by treatment with 50 mg/kg of Schisandrin A group; high, mice DN by treatment with 100 mg/kg of Schisandrin A group; control, control group; model, in vitro model group; low, in vitro model by 25 *μ*M of Schisandrin A group; med, in vitro model by 50 *μ*M of Schisandrin A group; high, in vitro model by 100 *μ*M of Schisandrin A group; ##*P* < 0.01 versus control group; ∗∗*P* < 0.01 versus STZ-induced mice DN group or in vitro model group.

Next, the experiment identified the biological function of Schisandrin A on oxidative stress and inflammation in model of DN. In mice model of DN or in vitro model of DN, these Schisandrin A could reduce inflammation factors release (IL-6, INF-*γ*, and TNF-*α*), also inhibited MDA activity level, and increased the level of SOD, CAT, and GHS levels (Figure 3). The results described above indicated that Schisandrin A exhibited a significant role in the anti-inflammation and antioxidant effects.

#### 3.3.1. Schisandrin A Reduced High Glucose-Induced Ferroptosis in Model of DN

**Figure 4:**
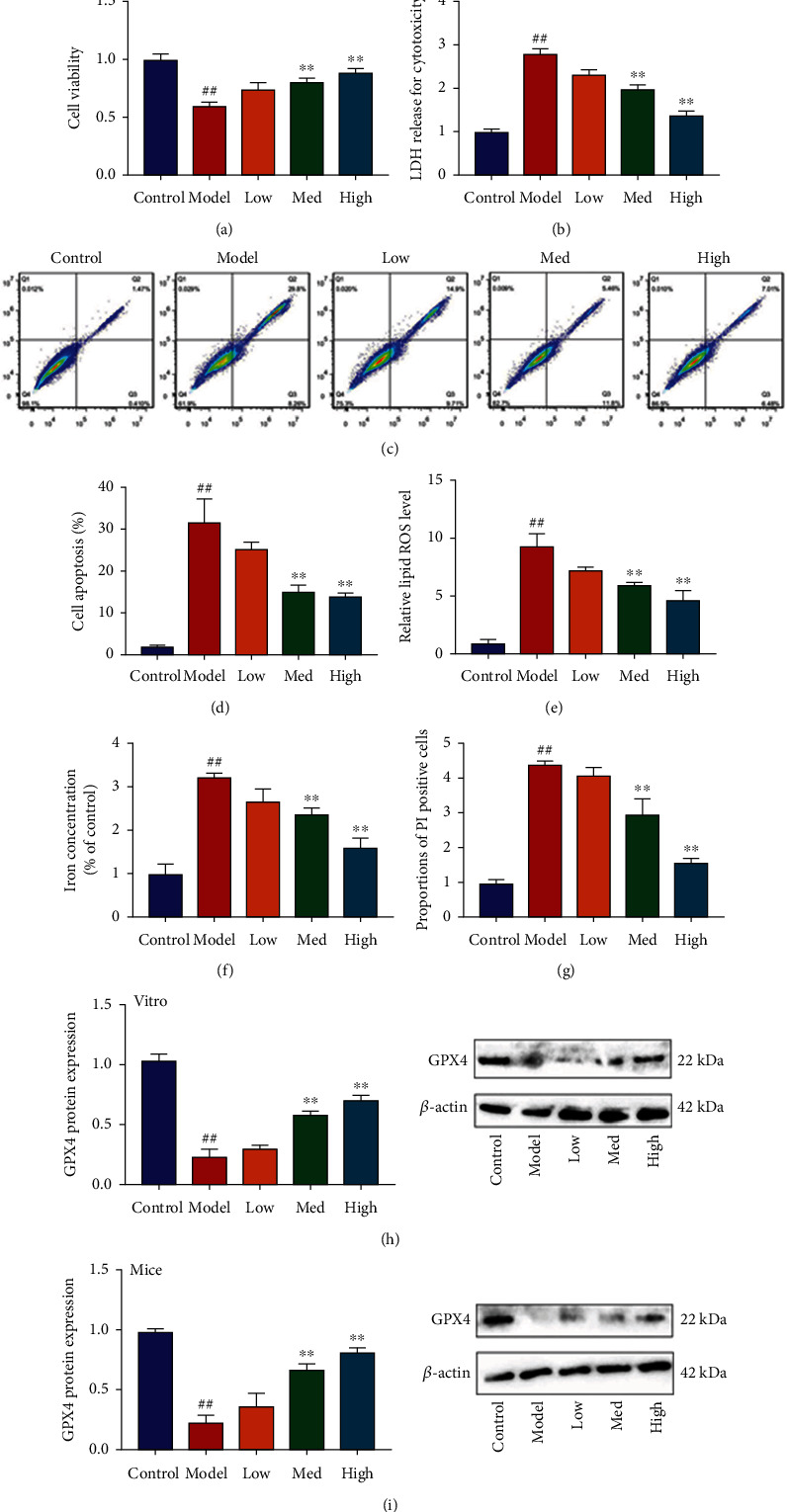
Schisandrin A reduced high glucose-induced ferroptosis in model of DN. (a) cell viability, (b) LDH activity level, (c and d) cell apoptosis, (e) lipid ROS levels, (f) iron concentration, (g) proportions of PI positive cells, (h) GPX4 protein expression in vitro model, and (i) GPX4 protein expression in mice model. Control, sham control mice group; model, STZ-induced mice DN group; low/med/high, mice DN by treatment with 25/50/100 mg/kg of Schisandrin A group; control, control group; model, in vitro model group; low/med/high, in vitro model by 25/50/100 *μ*M of Schisandrin A group; ##*P* < 0.01 versus control group; ∗∗*P* < 0.01 versus STZ-induced mice DN group or in vitro model group.

The study investigated the effects of Schisandrin A in high glucose-induced ferroptosis in model of DN. We found that Schisandrin A increased cell growth and inhibited LDH activity level and apoptosis rate in high glucose-induced in vitro model (Figures 4(a)–4(d)). Schisandrin A reduced lipid ROS levels and iron concentration and proportions of PI positive cells and inhibited calcein levels in high glucose-induced in vitro model (Figures 4(e)–4(g)). In addition, Schisandrin A suppressed GPX4 protein expressions in high glucose-induced in vitro model or mice model of DN (Figures 4(h) and 4(i)). Taken together, these results suggest that Schisandrin A aggravated high glucose-induced ferroptosis in model of DN.

#### 3.3.2. Schisandrin A Reduced ROS-Mediated Pyroptosis by Mitochondrial Damage in Model of DN

**Figure 5:**
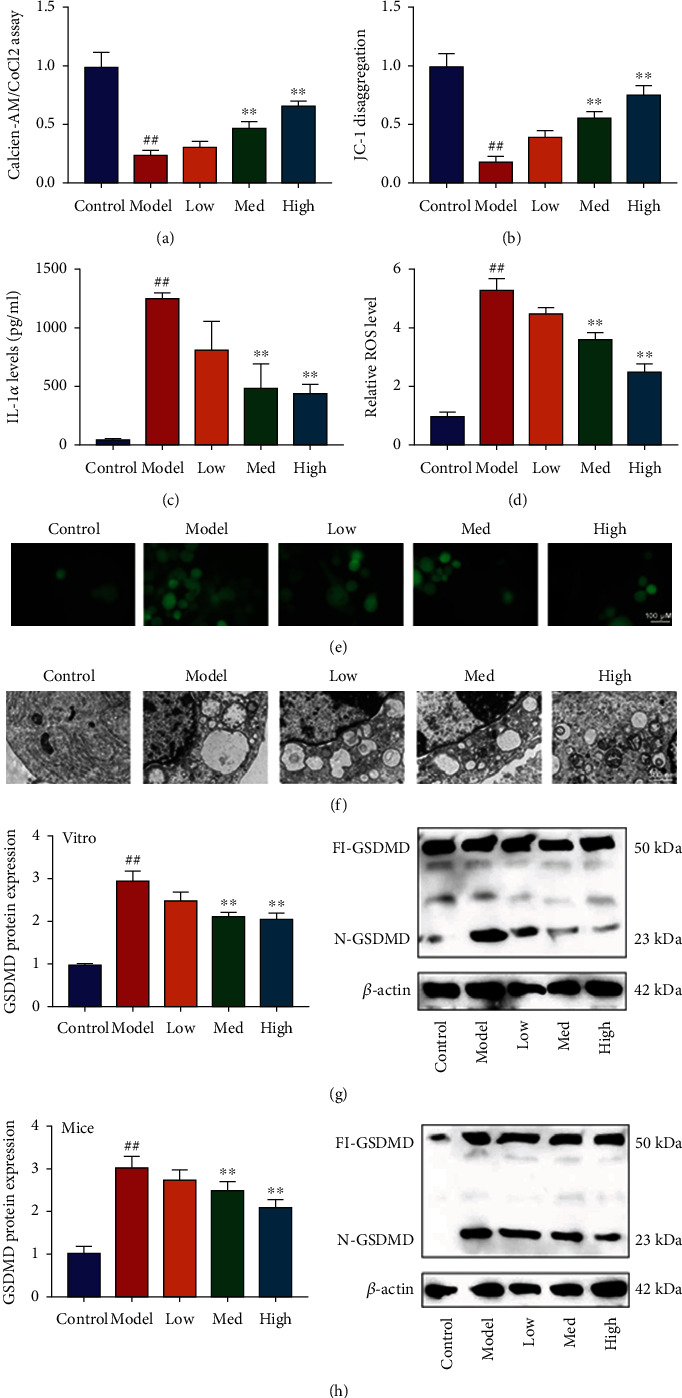
Schisandrin A reduced ROS-mediated pyroptosis by mitochondrial damage in model of DN. (a) Calcein-AM/CoCl2 assay, (b) JC-1 disaggregation, (c) IL-1*α* levels, (d and e) ROS production levels, (f) mitochondrial damage using electron microscope, (g) GSDMD protein expression in vitro model; and (h) GSDMD protein expression in mice model. Control, sham control mice group; model, STZ-induced mice DN group; low/med/high, mice DN by treatment with 25/50/100 mg/kg of Schisandrin A group; control, control group; model, in vitro model group; low/med/high, in vitro model by 25/50/100 *μ*M of Schisandrin A group; ##*P* < 0.01 versus control group; ∗∗*P* < 0.01 versus STZ-induced mice DN group or in vitro model group.

Our study explored that the effects and mechanism of Schisandrin A in model of DN. Schisandrin A increased MPT (calcein AM/CoCl2 assay) and mitochondrial damage (JC-1 disaggregation) and reduced IL-1*α* and ROS production levels in high glucose-induced in vitro model (Figures 5(a)–5(e)). Electron microscope showed that Schisandrin A decreased ROS-induced mitochondrial damage in high glucose-induced in vitro model (Figure 5(f)). Then, Schisandrin A suppressed GSDMD protein expression in high glucose-induced in vitro model or mice model of DN (Figures 5(g) and 5(h)). Taken together, our observations suggest that Schisandrin A contributes to the inhibition of ROS-mediated pyroptosis in model of DN by mitochondrial damage.

#### 3.3.3. Schisandrin A Suppressed TXNIP/NLRP3 In Vivo and In Vitro Model of DN

**Figure 6:**
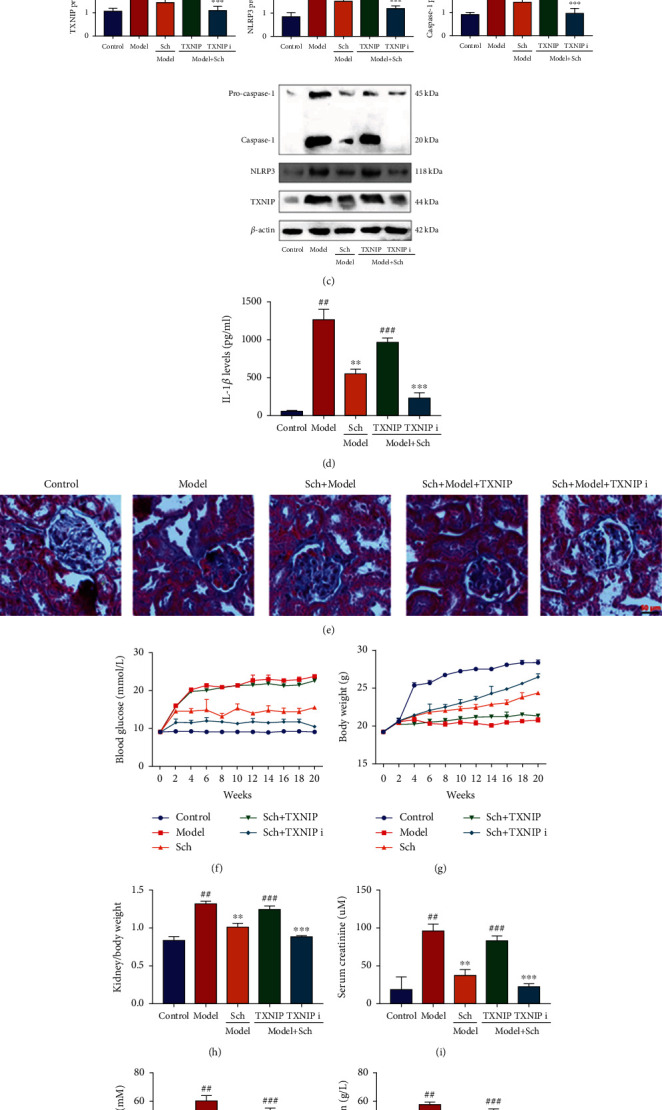
Schisandrin A suppressed TXNIP/NLRP3 in mice model of DN. (a) TXNIP, NLRP3 and caspase-1 protein expressions, (b) IL-1*β* levels in mice model of DN, (c) TXNIP, NLRP3 and caspase-1 protein expressions, (d) IL-1*β* levels in mice model of DN, (e) glomerulus injury (Masson staining), (f) blood glucose, (g) body weight, (h) kidney/body weight, (i) serum creatinine, (j) urea nitrogen, (k) urinary albumin levels, (l) water intake (24 h), and (m, n, and o) IL-6, INF-*γ*, and TNF-*α* in mice of DN. Control, sham control mice group; model, STZ-induced mice DN group; low/med/high, mice DN by treatment with 25/50/100 mg/kg of Schisandrin A group; Poly, 50 mg/kg of Schisandrin A group; TXNIP i, TXNIP inhibitor group; TXNIP, TXNIP up-regulation group; ##*P* < 0.01 versus control group; ∗∗*P* < 0.01 versus STZ-induced mice DN group; ∗∗∗*P* < 0.01 versus 50 mg/kg of Schisandrin A group; ###*P* < 0.01 versus 50 mg/kg of Schisandrin A group.

The study evaluated whether TXNIP/NLRP3 signaling pathway participated in the effects of Schisandrin A in vivo and in vitro model of DN. We found that Schisandrin A suppressed TXNIP, NLRP3, and caspase-1 protein expressions and decreased IL-1*β* levels in renal tissue of mice with DN (Figures 6(a) and 6(b)). So, these results suggest that TXNIP/NLRP3 signaling pathway might be one of the important targets for the effects of Schisandrin A in model of DN.

TXNIP virus induced TXNIP, NLRP3, and caspase-1 protein expressions, and increased IL-1*β* levels in renal tissue of mice with DN by treated with Schisandrin A (Figures 6(c) and 6(d)). TXNIP inhibitor (ruscogenin) also suppressed TXNIP, NLRP3, and caspase-1 protein expressions and decreased IL-1*β* levels in renal tissue of mice with DN by treated with Schisandrin A (Figures 6(c) and 6(d)). TXNIP virus increased glomerulus injury and blood glucose, reduced body weight, heightened kidney/body weight and serum creatinine, promoted urea nitrogen and urinary albumin levels, and enhanced inflammation factors release in mice with DN by treated with Schisandrin A (Figures 6(e)–6(o)). TXNIP inhibitor (ruscogenin) promoted the effects of Schisandrin A on glomerulus injury, blood glucose, body weight, kidney/body weight, serum creatinine, urea nitrogen, urinary albumin levels, and inflammation factors release in mice with DN (Figures 6(e)–6(o)).

TXNIP virus heightened RKA level and periostin mRNA expression; decreased RKVd, RKVm, and RKVs levels; and reduced E-cadherin mRNA expression in mice with DN by treated with Schisandrin A (Figures S1A–S1F). TXNIP inhibitor (ruscogenin) reduced RKA level and periostin mRNA expression; increased RKVd, RKVm, and RKVs levels; and promoted E-cadherin mRNA expression in mice with DN by treated with Schisandrin A (Figures S1A–S1F).

Next, in vitro model of DN, Schisandrin A suppressed TXNIP, NLRP3, and caspase-1 protein expressions and decreased IL-1*β* levels (Figures S2A and S2B). TXNIP plasmid induced TXNIP, NLRP3, and caspase-1 protein expressions and increased IL-1*β* levels in vitro model of DN by treated with Schisandrin A (Figures S2C and S2D). Si-TXNIP mimics suppressed TXNIP, NLRP3, and caspase-1 protein expressions and decreased IL-1*β* levels in vitro model of DN by treated with Schisandrin A (Figures S2C and S2D). TXNIP plasmid reduced cell growth, increased LDH activity levels and the proportions of PI positive cells, inhibited JC-1 disaggregation and MPT (calcein AM/CoCl2 assay), and induced IL-1*α* levels and GSDMD protein expressions in vitro model of DN by treated with Schisandrin A (Figures S2E–S2K). Meanwhile, TXNIP plasmid suppressed E-cadherin mRNA expression and induced periostin mRNA expression and inflammation factors release in vitro model of DN by treated with Schisandrin A (Figures S1G–S1K). Si-TXNIP mimics promoted cell growth, decreased LDH activity levels and the proportions of PI positive cells, increased JC-1 disaggregation and MPT (calcein AM/CoCl2 assay), and inhibited IL-1*α* levels and GSDMD protein expressions in vitro model of DN by treated with Schisandrin A (Figures S2E–S2K). Additionally, si-TXNIP mimics induced E-cadherin mRNA expression and suppressed periostin mRNA expression and inflammation factors release in vitro model of DN by treated with Schisandrin A (Figures S1G–S1K). Collectively, our findings suggest that TXNIP-mediated NLRP3-induced pyroptosis is essential for Schisandrin A-presented DN.

#### 3.3.4. Schisandrin A Directly Targeted AdipoR1 Protein

**Figure 7:**
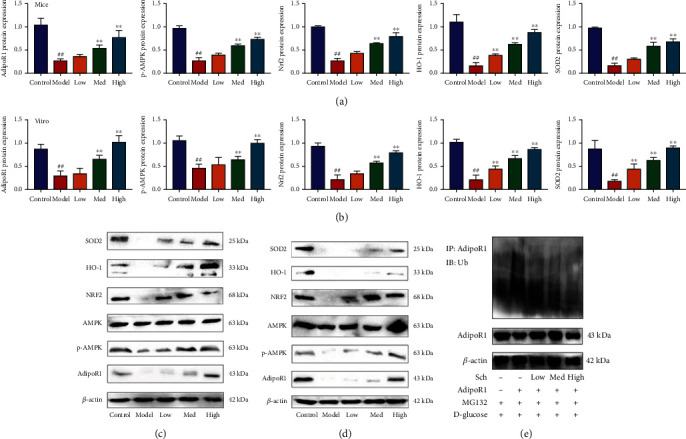
Schisandrin A induced AdipoR1 protein and suppressed AdipoR1 ubiquitination. (a and c) AdipoR1, p-AMPK, Nrf2, HO-1, and SOD2 protein expression in mice model; (b and d) AdipoR1, p-AMPK, Nrf2, HO-1, and SOD2 protein expression in vitro model; and AdipoR1 ubiquitination in vitro model (e). Control, sham control mice group; model, STZ-induced mice DN group; low/med/high, mice DN by treatment with 25/50/100 mg/kg of Schisandrin A group; ##*P* < 0.01 versus control group; ∗∗*P* < 0.01 versus STZ-induced mice DN group. Control, sham control mice group; model, in vitro model of DN group; low/med/high, in vitro model of DN by treatment with 25/50/100 *μ*M of Schisandrin A group; ##*P* < 0.01 versus control group; ∗∗*P* < 0.01 versus in vitro model group.

The experiment evaluated the mechanism of Schisandrin A in model of DN. We found that Schisandrin A induced AdipoR1, p-AMPK, Nrf2, HO-1, and SOD2 protein expressions in renal tissue of mice with DN (Figures 7(a) and 7(c)). In vitro model, we also found that Schisandrin A induced AdipoR1, p-AMPK, Nrf2, HO-1, and SOD2 protein expressions (Figures 7(b) and 7(d)). Schisandrin A reduced LPS + ATP-induced AdipoR1 ubiquitination in vitro model (Figure 7(e)).

**Figure 8:**
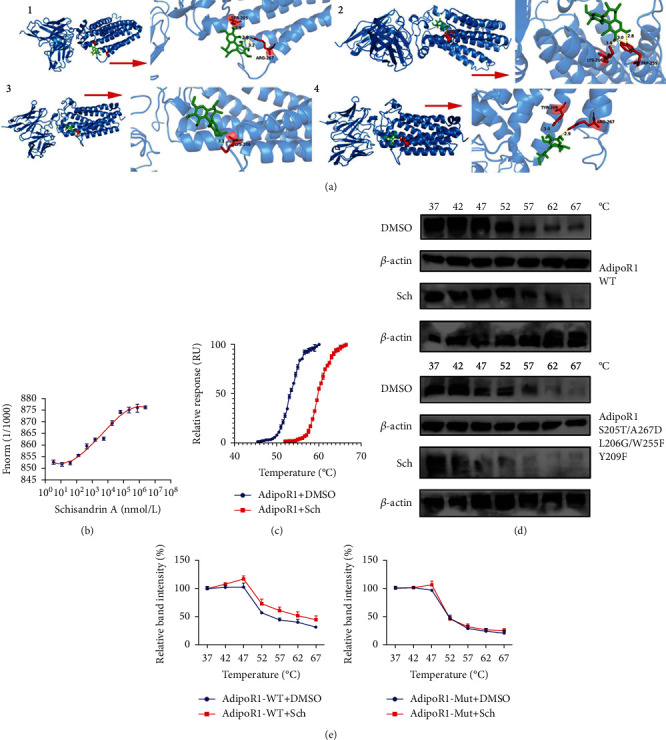
Schisandrin A directly targeted AdipoR1 protein. (a) The 3D image revealed that Schisandrin A bond to the binding pocket and formed with AdipoR1. (b) Microscale thermophoresis (MST) of Schisandrin A and AdipoR1 incubated with Schisandrin A. (c) TSA results in the presence or absence of Schisandrin A. (d) The thermal stability of WT AdipoR1 and Mut AdipoR1 plasmid after treatment with Schisandrin A using CETSA. (e) CETSA curve and the thermal stability to reach 50% of temperature (Tm50) value.

Drug and protein linkage analysis showed Schisandrin A linkage AdipoR1 (Figure 8(a)). Schisandrin A affects the thermophoretic motion of AdipoR1; upon binding with Schisandrin A, the melting temperature of AdipoR1 was elevated from ~55 to ~60°C (Figures 8(b) and 8(c)). CETSA with HEK293T cells demonstrated that Schisandrin A largely improved the thermal stability of exogenous WT AdipoR1, while Schisandrin A did not change the thermal stability of Mut AdipoR1, indicating that SER-205, ARG-267, LYS-206, TRP-255, and TYR-209 might be responsible for the interaction between AdipoR1 and Schisandrin A (Figures 8(d) and 8(e)). We considered that AdipoR1 might be a one target spot for the effects of Schisandrin A in model of DN.

#### 3.3.5. *Schisandrin* A Activated AdipoR1/AMPK In Vivo And In Vitro Model of DN

**Figure 9:**
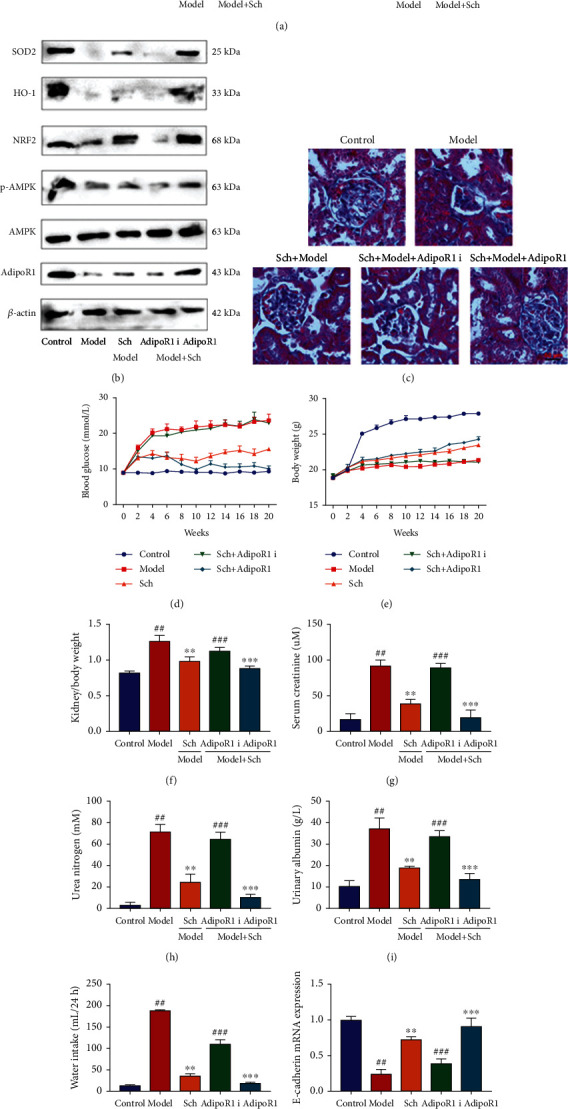
The regulation of AdipoR1 affected the effects of Schisandrin A in mice model of DN. (a and b) AdipoR1, p-AMPK, Nrf2, HO-1, and SOD2 protein expression in mice model; (c) glomerulus injury (Masson staining); (d) blood glucose; (e) body weight; (f) kidney/body weight; (g) serum creatinine; (h) urea nitrogen; (i) urinary albumin levels; (j) water intake (24 h); (k) E-cadherin mRNA expression; and (l) periostin mRNA expression in mice of DN. Control, sham control mice group; model, STZ-induced mice DN group; low/med/high, mice DN by treatment with 25/50/100 mg/kg of Schisandrin A group; Poly, 50 mg/kg of Schisandrin A group; AdipoR1 i, sh-AdipoR1 group; AdipoR1, AdipoR1 Agonist group; ##*P* < 0.01 versus control group; ∗∗*P* < 0.01 versus STZ-induced mice DN group; ∗∗∗*P* < 0.01 versus 50 mg/kg of Schisandrin A group; ###*P* < 0.01 versus 50 mg/kg of Schisandrin A group.

To determine the role of AdipoR1 in the effects of Schisandrin A in model of DN, we regulated the expression of AdipoR1 in mice with DN by Schisandrin A. Sh-AdipoR1 reduced AdipoR1, p-AMPK, Nrf2, HO-1, and SOD2 protein expressions in renal tissue of mice with DN by treated with Schisandrin A (Figures 9(a) and 9(b)). AdipoR1 agonist (gramine) induced AdipoR1, p-AMPK, Nrf2, HO-1, and SOD2 protein expressions in renal tissue of mice with DN by treated with Schisandrin A (Figures 9(a) and 9(b)). Sh-AdipoR1 increased glomerulus injury and blood glucose, reduced body weight, heightened kidney/body weight and serum creatinine, promoted urea nitrogen and urinary albumin levels, suppressed E-cadherin mRNA expression, and induced periostin mRNA expression in mice with DN by treated with Schisandrin A (Figures 9(c)–9(l)). Additionally, AdipoR1 agonist decreased glomerulus injury and blood glucose, promoted body weight, heightened kidney/body weight and serum creatinine, reduced urea nitrogen and urinary albumin levels, induced E-cadherin mRNA expression, and suppressed periostin mRNA expression in mice with DN by treated with Schisandrin A (Figures 9(c)–9(l)).

Conversely, we next evaluated that the mechanism of Schisandrin A in model of DN using in vitro model. AdipoR1 plasmid increased induced AdipoR1, p-AMPK, Nrf2, HO-1, and SOD2 protein expressions in vitro model by treated with Schisandrin A (Figure S3A). Si-AdipoR1 mimics suppressed AdipoR1, p-AMPK, Nrf2, HO-1, and SOD2 protein expressions in vitro model by treated with Schisandrin A (Figure S3A). AdipoR1 plasmid could increase the effects of Schisandrin A on cell growth, LDH activity level, the proportions of PI positive cells, JC-1 disaggregation, MPT (calcein AM/CoCl2 assay), iron concentration, lipid ROS levels, IL-1*α* levels, SOD activity levels, and MDA levels in vitro model by treated with Schisandrin A (Figures S3B–S3L). AdipoR1 plasmid induced GPX4 protein expression and suppressed GSDMD protein expression in vitro model by treated with Schisandrin A (Figure S3M). Si-AdipoR1 reduced the effects of Schisandrin A on cell growth, LDH activity level, the proportions of PI positive cells, JC-1 disaggregation, MPT (calcein AM/CoCl2 assay), iron concentration, lipid ROS levels, IL-1*α* levels, SOD activity levels, and MDA levels in vitro model by treated with Schisandrin A (Figures S3B–S3L). Si-AdipoR1 suppressed GPX4 protein expression and induced GSDMD protein expression in vitro model by treated with Schisandrin A (Figure S3M). The results described above indicated that Schisandrin A activated AdipoR1/AMPK signaling pathway to the inhibition of ROS-mediated pyroptosis by mitochondrial damage in vivo and in vitro model of DN.

Additionally, sh-AdipoR1 induced TXNIP, NLRP3, and caspase-1 protein expressions and increased IL-1*β* levels in renal tissue of mice with DN by treated with Schisandrin A (Figures S4A–S4D). AdipoR1 agonist (gramine) suppressed TXNIP, NLRP3, and caspase-1 protein expressions, and decreased IL-1*β* levels in renal tissue of mice with DN by treated with Schisandrin A (Figures S4A–S4D). Then, sh-AdipoR1 induced TXNIP, NLRP3, and caspase-1 protein expressions and increased IL-1*β* levels in vitro model of DN by treated with Schisandrin A (Figures S4E–S4H). Furthermore, AdipoR1 plasmid suppressed TXNIP, NLRP3, and caspase-1 protein expressions and increased IL-1*β* levels in vitro model of DN by treated with Schisandrin A (Figures S4E–S4H).

Sh-AdipoR1 induced inflammation factors release and MDA levels, inhibited SOD activity level, suppressed GPX4 protein expression, and heightened GSDMD protein expression in renal tissue of mice with DN by treated with Schisandrin A (Figures S4I–S4M and S4Q). AdipoR1 agonist (gramine) reduced inflammation factors release and MDA levels, and increased SOD activity level, induced GPX4 protein expression, and suppressed GSDMD protein expression in renal tissue of mice with DN by treated with Schisandrin A (Figures S4I–S4M and S4Q). Furthermore, sh-AdipoR1 increased inflammation factors release in vitro model of DN by treated with Schisandrin A (Figures S4N–S4P). AdipoR1 plasmid reduced inflammation factors release in vitro model of DN by treated with Schisandrin A (Figures S4N–S4P). These results indicated that Schisandrin A suppressed TXNIP/NLRP3 signaling pathway in mice model of DN.

## 4. Discussion

With the adult diabetes prevalence rate, China has become one of the countries with the fastest growth rate of diabetes prevalence [22]. Since type 2 diabetes is an important cause of end-stage renal disease, the early diagnosis and treatment of type 2 diabetes with renal damage have been a hotspot for medical workers at home and abroad [23, 24]. Dong et al. validated that Schisandrin A protected high glucose-induced cell injury [25]. However, our experiment first found that Schisandrin A reduced acute kidney injury in model of DN through the inhibition of oxidative stress and inflammation. Kwon et al. concluded that Schisandrin A suppresses LPS-induced inflammation in RAW 264.7 macrophages [26]. So, these results indicated that Schisandrin A reduced oxidative stress and inflammation to present acute kidney injury in model of DN. However, Schisandrin A affects insulin resistance in model of DN, or other complications of diabetes were unclear. We will research Schisandrin A present DN through the regulation of insulin resistance and its possible mechanisms in further experiment.

Adiponectin is a cytokine secreted by differentiated and mature adipocytes, and its biological function is realized through its receptors AdipoR1 and AdipoR2 [27]. Adiponectin receptors are mainly expressed in target organs that are acted on by insulin, such as liver, skeletal muscle, and pancreatic islet cells [28]. According to related studies, both AdipoR1 and R2 can be expressed in pancreatic islet cells, with AdipoR1 being the main expression [29, 30]. In the present study, Schisandrin A activated AdipoR1 expression in vivo and in vitro model of DN. Therefore, AdipoR1 might be a one target spot for the effects of Schisandrin A in model of DN.

AdipoR1 works by activating the AMPK pathway [30]. Under high glucose condition, adiponectin binds to cell membrane surface receptors to activate AMPK pathway and significantly increase insulin secretion, thereby lowering blood sugar [31]. AMPK, also known as “energy receptor,” participates in regulating cell metabolism [31]. Through the ratio of AMP/ROS, the activity is controlled, and the body energy is kept in balance [31]. AMPK is involved in regulating the terminal link of insulin secretion by pancreatic *β* cells. In pancreatic *β* cells, glucose can change the level of adenylate in the cell through oxidative metabolism, which in turn affects AMPK activity [30]. Xu et al. suggest that Schisandrin A protects against lipopolysaccharide-induced mastitis by activating AMPK/Nrf2 signaling pathway [32]. This experiment showed that Schisandrin A induced p-AMPK, Nrf2, HO-1, and SOD2 protein expressions in model of DN by AdipoR1. These data implied a pivotal role of AdipoR1/AMPK signaling pathway in the anti-inflammation and antioxidant effects of Schisandrin A in DN.

ROS is considered to be the common signal and regulatory center of NLRP3 inflammasome activation. For the ROS involved in the activation of the NLRP3 inflammasome, the mitochondrial source is currently more recognized, namely, mitochondrial ROS (mtROS) [33, 34]. Studies have confirmed that there is a close correlation between mitochondrial dysfunction and the activation of NLRP3 inflammasome [17]. Under the stimulation of various NLRP3 agonists, mitochondria become dysfunctional, and the production of mtROS increases, which leads to the formation of NLRP3 inflammasomes and the release of inflammatory mediators [35]. Hyun Choi et al. indicate that Schisandrin A reduced oxidative stress-induced DNA damage and apoptosis in C2C12 cells by the inhibition of ROS generation [36]. Additionally, we showed that Schisandrin A reduced ROS-mediated pyroptosis by mitochondrial damage in model of DN, further supporting that Schisandrin A lessened ROS-mediated pyroptosis in DN through targeting AdipoR1/AMPK signaling pathway.

The NLRP3 inflammasome is the activation platform of the caspase-1 molecule, which regulates the secretion and maturation of various inflammatory factors, such as IL-18 and IL-1*β*, and participates in the natural immune response [37, 38]. According to recent studies, the NLRP3 inflammasome plays an important role in the inflammatory response of kidney disease [39]. The increase of IL-1*β* level is an important risk factor for the progression of type 2 diabetes and insulin resistance [40, 41]. By inhibiting its activation, the inflammatory response of rat renal tissue can be significantly weakened, and the renal function improved [40, 41]. Gao et al. showed that Schisandrin A suppressed pyroptosis by inhibiting NLRP3 inflammasome in THP-1 cells [42]. In this paper, Schisandrin A suppressed TXNIP/NLRP3 in vivo and in vitro model of DN by the activation of AdipoR1/AMPK signaling pathway. These results proved that TXNIP/NLRP3 regulated the anti-inflammation effects of Schisandrin A on inflammation in model of DN.

Pyrolysis can induce the occurrence of DN inflammation in spontaneous inflammatory diseases through exogenous infection and endogenous injury signals [43]. The pathways to activate inflammasome include abnormal autophagy, abnormal production of mitochondrial ROS, and some metal state imbalance [44]. As mentioned earlier, phagolysosome disorders can also activate the NLRP3 inflammasome [45, 46]. Gao et al. showed that Schisandrin A suppressed pyroptosis by inhibiting NLRP3 inflammasome in THP-1 cells [42]. In the present study, we documented that the regulation of TXNIP/NLRP3 adjusted the inflammation effects of Schisandrin A on pyroptosis in model of DN. These data uncovered that the TXNIP-mediated NLRP3-induced pyroptosis is essential for Schisandrin A-presented DN.

Iron death is mainly due to the abnormal increase of intracellular “iron” dependent on lipid oxygen free radicals and the imbalance of redox homeostasis [47]. Under the stimulation of high glucose, the volume of podocyte mitochondria decreased under the transmission electron microscope, the density of the double membrane increased, and the mitochondrial crest decreased or disappeared [48]. The depletion of GSH in podocytes and the obvious increase of ROS levels may indicate the iron death [49]. Zhang et al. indicated that Schisandrin B reduced lipoperoxidative damage by iron/cysteine [36]. Furthermore, this study displayed that Schisandrin A reduced high glucose-induced ferroptosis in model of DN, indicating that Schisandrin A functioned to reduce ferroptosis by AdipoR1/AMPK signaling pathway in model of DN.

Nrf2 is a transcription factor that initiates the endogenous antioxidant response element, which undergoes nuclear transport to exert its antioxidative stress ability when stimulated by external reactive oxygen species [50, 51]. Nuclear transfer of Nrf2 activates the transcription of a large number of downstream antioxidant enzyme genes such as HO-1 and GPX4, thereby reducing iron death in podocytes [52, 53]. Ni et al. showed that the Schisandrin A restrains osteoclastogenesis by Nrf2 signaling [54]. In this study, we validated that Schisandrin A induced Nrf2/HO-1/GPX4 expression in model of DN. Thus, we suggested that the AdipoR1/AMPK/Nrf2/HO-1/GPX4 axis plays a critical pathogenic role in the effects of Schisandrin A on ferroptosis in model of DN.

**Figure 10:**
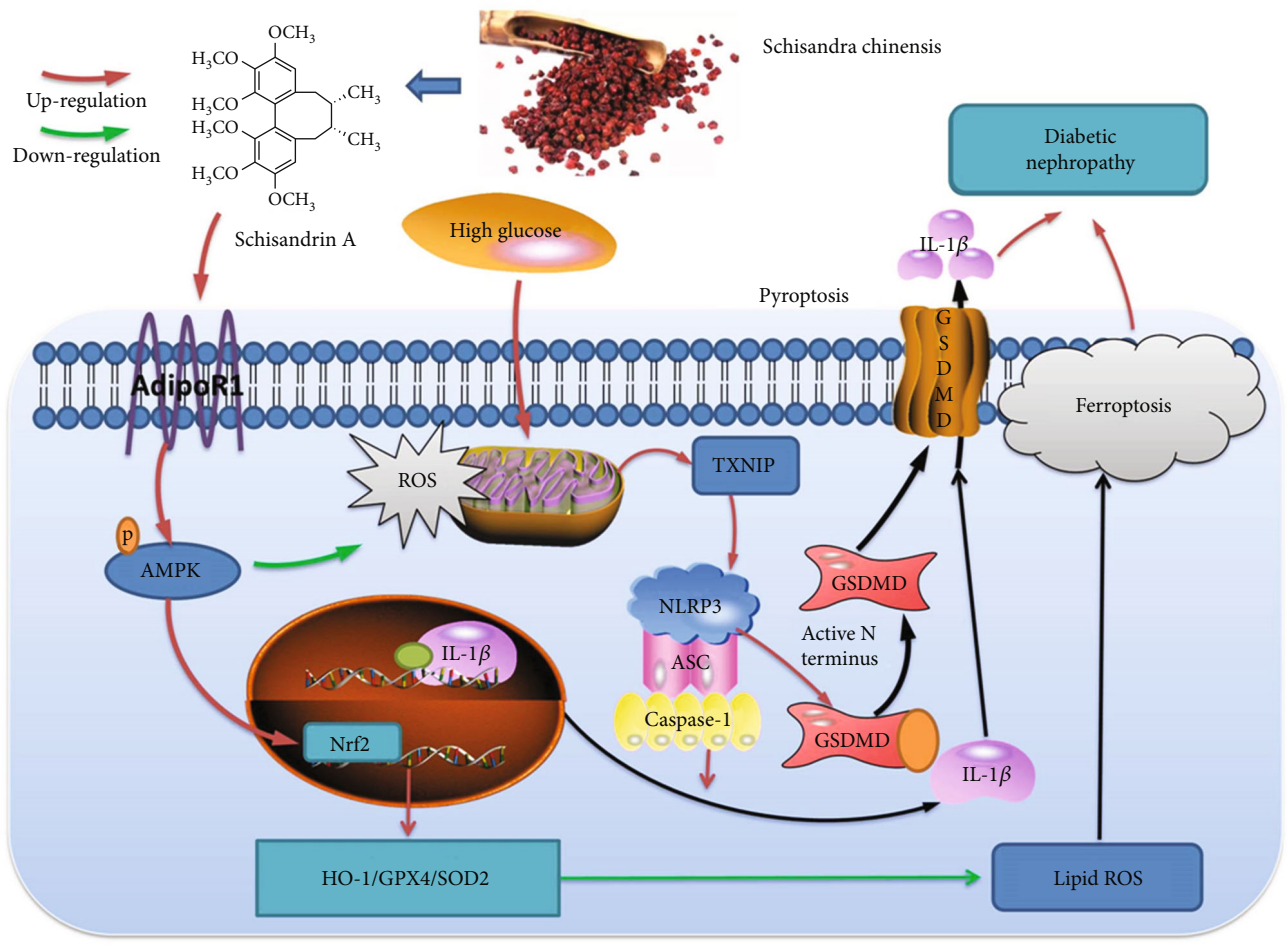
Schisandrin A from *Schisandra chinensis* attenuates ferroptosis and NLRP3 inflammasome-mediated pyroptosis in diabetic nephropathy through AMPK-ROS/mitochondrial damage by AdipoR1 ubiquitination.

In conclusion, our study provided direct evidence that Schisandrin A from *Schisandra chinensis* attenuates ferroptosis and NLRP3 inflammasome-mediated pyroptosis in DN by AdipoR1/AMPK-ROS/mitochondrial damage (Figure 10). Additionally, this study confirmed that Schisandrin A is a possible therapeutic option for DN or other diabetes, but the specific mechanism needs to be investigated further.

## Data Availability

The datasets used and/or analyzed of this study are from corresponding author upon reasonable request.

## Abbreviations

DN: Diabetic nephropathy
STZ: Streptozotocin
ROS: Reactive oxygen species
ESRD: End-stage renal disease
HFD: High-fat diet
MST: Microscale thermophoresis
TSA: Thermal shift assay
CETSA: Cellular thermal shift assay
GSDMD: Gasdermin D
IL-1*β*: Interleukin-1beta
HRGECs: Human renal glomerular endothelial cells
mtROS: Mitochondrial ROS
NLRP3: NACHT, LRR, and PYD domains-containing protein 3.

## Supplementary Materials

Supplementary 1 Figure S1. The regulation of TXNIP affected the effects of Schisandrin A on diabetic nephropathy in model of DN. (A, B, C, and D) RKA, RKVd, RKVm, and RKVs levels in mice model; (E) E-cadherin mRNA expression; (F) periostin mRNA expression in mice of DN; (G) E-cadherin mRNA expression; (H) periostin mRNA expression in vitro model of DN; (I, J, and K) IL-6, INF-*γ*, and TNF-*α* in vitro model; control, sham control mice group; model, STZ-induced mice DN group; Poly, 50 mg/kg of Schisandrin A group; TXNIP i, TXNIP inhibitor group; TXNIP, TXNIP up-regulation group; TXNIP, overexpression of TXNIP group; Si-TXNIP, down-regulation of TXNIP group. ##*P* < 0.01 versus control group; ∗∗*P* < 0.01 versus STZ-induced mice DN group; ∗∗∗*P* < 0.01 versus 50 mg/kg of Schisandrin A group; ###*P* < 0.01 versus 50 mg/kg of Schisandrin A group.

Supplementary 2 Figure S2. Schisandrin A suppressed TXNIP/NLRP3 in vitro model of DN. (A) TXNIP, NLRP3, and caspase-1 protein expressions; (B) IL-1*β* levels in vitro model of DN; (C) TXNIP, NLRP3, and caspase-1 protein expressions; (D) IL-1*β* levels in vitro model of DN; (E) cell viability; (F) LDH activity level; (G) proportions of PI positive cells; (H) JC-1 disaggregation; (I) calcein-AM/CoCl2 assay; (J) IL-1*α* levels; and (K) GSDMD protein expression in vitro model. Control, sham control mice group; model, in vitro model of DN group; low/med/high, in vitro model of DN by treatment with 25/50/100 *μ*M of Schisandrin A group; Poly, 50 *μ*M of Schisandrin A group; TXNIP, overexpression of TXNIP group; Si-TXNIP, down-regulation of TXNIP group. ##*P* < 0.01 versus control group; ∗∗*P* < 0.01 versus in vitro model group; ∗∗∗*P* < 0.01 versus 50 mg/kg of Schisandrin A group; ###*P* < 0.01 versus 50 *μ*M of Schisandrin A group.

Supplementary 3 Figure S3. Schisandrin A activated AdipoR1/AMPK in vitro model of DN (A) AdipoR1, p-AMPK, Nrf2, HO-1, and SOD2 protein expression in mice model, (B) cell viability, (C) LDH activity level, (D) proportions of PI positive cells, (E) JC-1 disaggregation, (F) calcein-AM/CoCl2 assay, (G) IL-1*α* levels, (H) iron concentration, (I) ROS protein levels, (J) lipid ROS levels, (K) SOD level, (L) MDA level, and (M) GPX4/GSDMD protein expression in vitro model. Control, sham control mice group; model, in vitro model of DN group; low/med/high, in vitro model of DN by treatment with 25/50/100 *μ*M of Schisandrin A group; Poly, 50 *μ*M of Schisandrin A group; AdipoR1, overexpression of AdipoR1 group; Si-AdipoR1, down-regulation of AdipoR1 group. ##*P* < 0.01 versus control group; ∗∗*P* < 0.01 versus in vitro model group; ∗∗∗*P* < 0.01 versus 50 mg/kg of Schisandrin A group; ###*P* < 0.01 versus 50 *μ*M of Schisandrin A group.

Supplementary 4 Figure S4. The regulation of AdipoR1 affected the effects of Schisandrin A on TXNIP/NLRP3 in model of DN. (A, B, and C) TXNIP, NLRP3, and caspase-1 protein expressions; (D) IL-1*β* levels in mice model of DN; (E, F, and G) TXNIP, NLRP3 and caspase-1 protein expressions; (H) IL-1*β* levels in mice model of DN; (I, J, K, L, and M) IL-6, INF-*γ*, TNF-*α*, MDA, and SOD levels in mice model of DN; (N, O, and P) IL-6, INF-*γ*, and TNF-*α* in vitro model; and (Q) GPX4 and GSDMD protein expression in mice model of DN. Control, sham control mice group; model, STZ-induced mice DN group; low/med/high, mice DN by treatment with 25/50/100 mg/kg of Schisandrin A group; Poly, 50 mg/kg of Schisandrin A group; AdipoR1 i, sh-AdipoR1 group; AdipoR1, AdipoR1 agonist group; ##*P* < 0.01 versus control group; ∗∗*P* < 0.01 versus STZ-induced mice DN group; ∗∗∗*P* < 0.01 versus 50 mg/kg of Schisandrin A group; ###*P* < 0.01 versus 50 mg/kg of Schisandrin A group.
