# Supplementary figures and images for: Schisandrin A from Schisandra chinensis Attenuates Ferroptosis and NLRP3 Inflammasome-Mediated Pyroptosis in Diabetic Nephropathy through Mitochondrial Damage by AdipoR1 Ubiquitination
Source: Oxid Med Cell Longev. 2022 Aug 11;2022:5411462. doi: 10.1155/2022/5411462 (PMC9391610; doi:10.1155/2022/5411462)

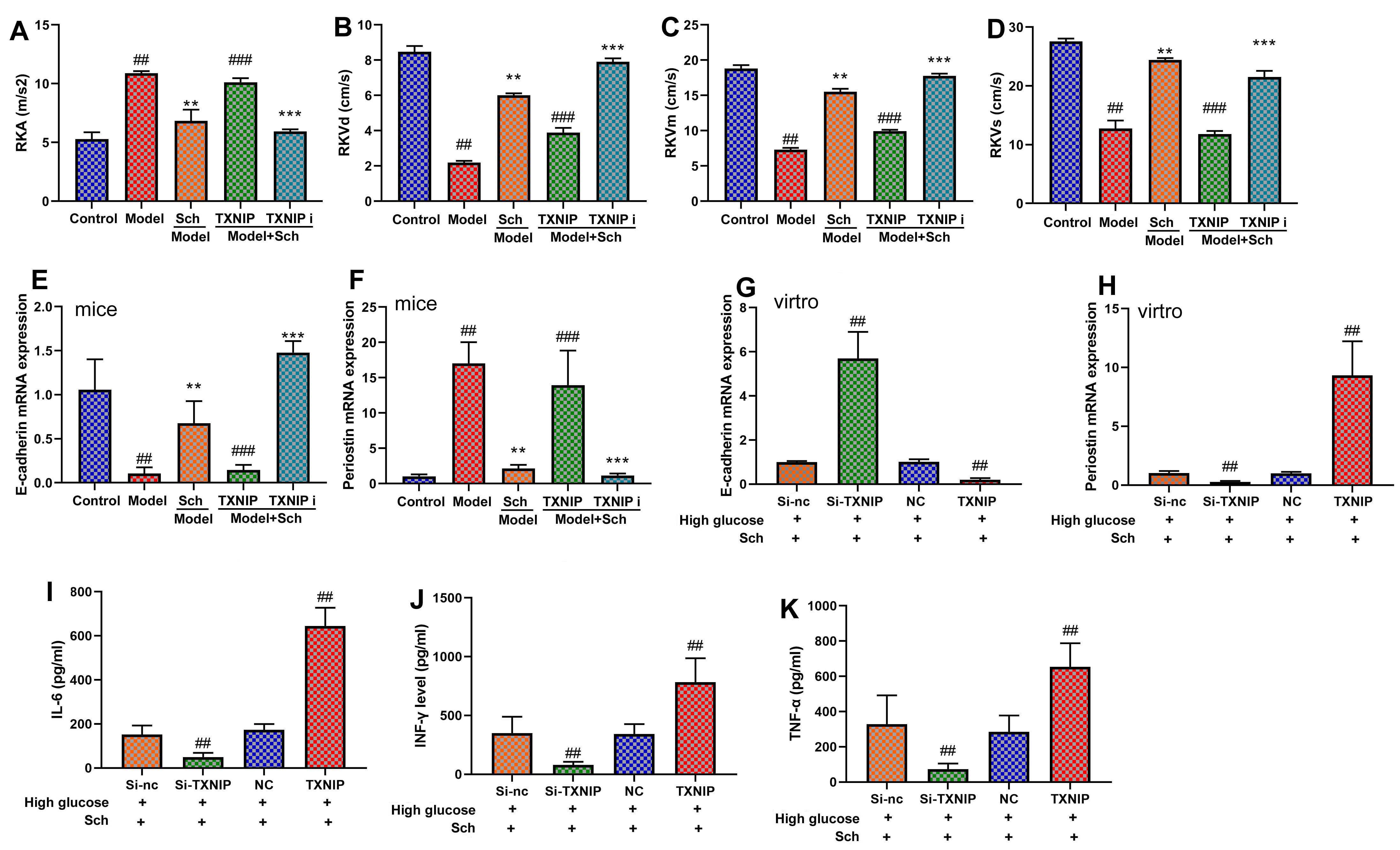

Supplement: Supplementary 1 — Figure S1. The regulation of TXNIP affected the effects of Schisandrin A on diabetic nephropathy in model of DN. (A, B, C, and D) RKA, RKVd, RKVm, and RKVs levels in mice model; (E) E-cadherin mRNA expression; (F) periostin mRNA expression in mice of DN; (G) E-cadherin mRNA expression; (H) periostin mRNA expression in vitro model of DN; (I, J, and K) IL-6, INF-γ, and TNF-α in vitro model; control, sham control mice group; model, STZ-induced mice DN group; Poly, 50 mg/kg of Schisandrin A group; TXNIP i, TXNIP inhibitor group; TXNIP, TXNIP up-regulation group; TXNIP, overexpression of TXNIP group; Si-TXNIP, down-regulation of TXNIP group. ##P < 0.01 versus control group; ∗∗P < 0.01 versus STZ-induced mice DN group; ∗∗∗P < 0.01 versus 50 mg/kg of Schisandrin A group; ###P < 0.01 versus 50 mg/kg of Schisandrin A group. [file 5411462.f1.jpg]

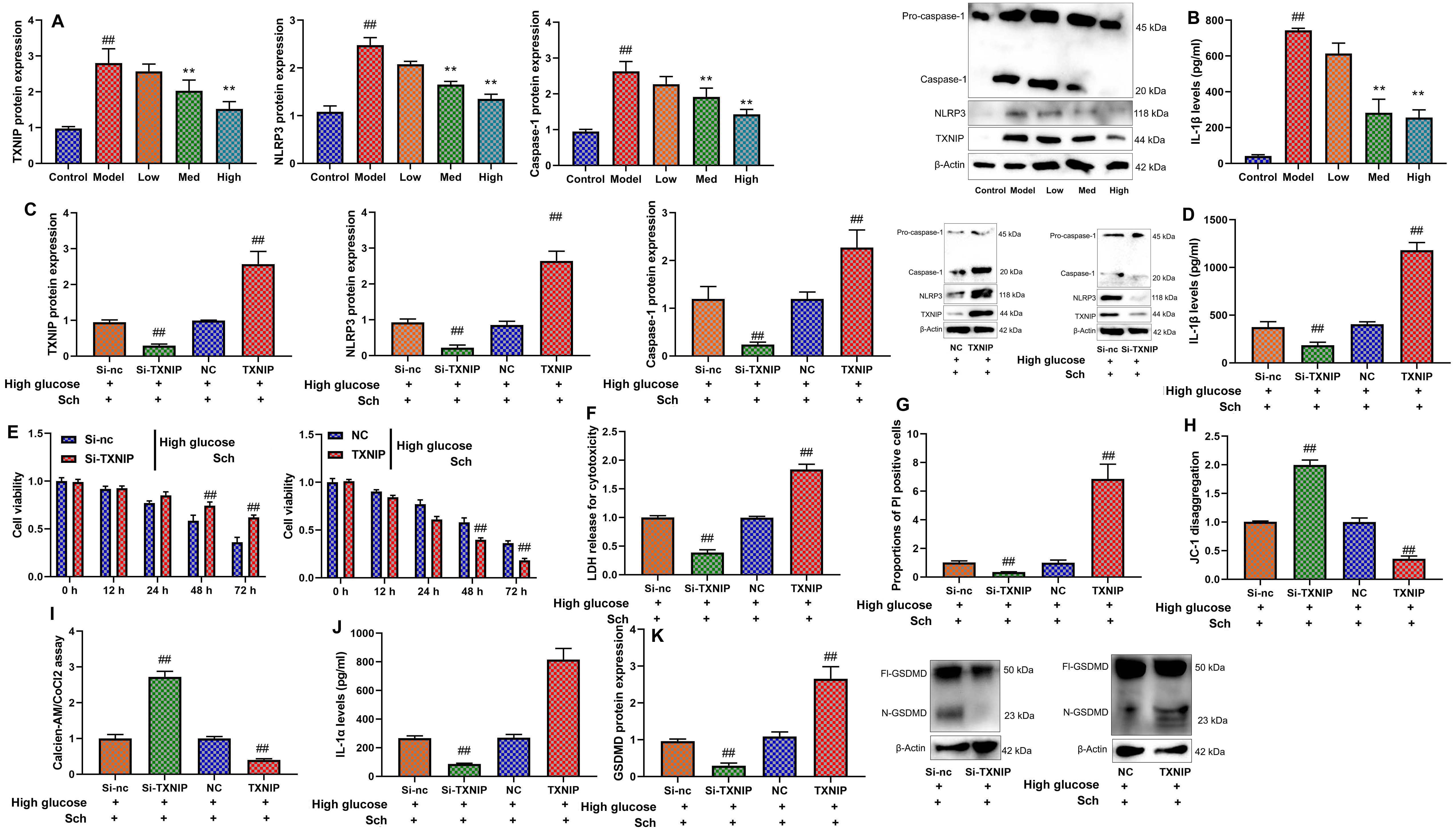

Supplement: Supplementary 2 — Figure S2. Schisandrin A suppressed TXNIP/NLRP3 in vitro model of DN. (A) TXNIP, NLRP3, and caspase-1 protein expressions; (B) IL-1β levels in vitro model of DN; (C) TXNIP, NLRP3, and caspase-1 protein expressions; (D) IL-1β levels in vitro model of DN; (E) cell viability; (F) LDH activity level; (G) proportions of PI positive cells; (H) JC-1 disaggregation; (I) calcein-AM/CoCl2 assay; (J) IL-1α levels; and (K) GSDMD protein expression in vitro model. Control, sham control mice group; model, in vitro model of DN group; low/med/high, in vitro model of DN by treatment with 25/50/100 μM of Schisandrin A group; Poly, 50 μM of Schisandrin A group; TXNIP, overexpression of TXNIP group; Si-TXNIP, down-regulation of TXNIP group. ##P < 0.01 versus control group; ∗∗P < 0.01 versus in vitro model group; ∗∗∗P < 0.01 versus 50 mg/kg of Schisandrin A group; ###P < 0.01 versus 50 μM of Schisandrin A group. [file 5411462.f2.jpg]

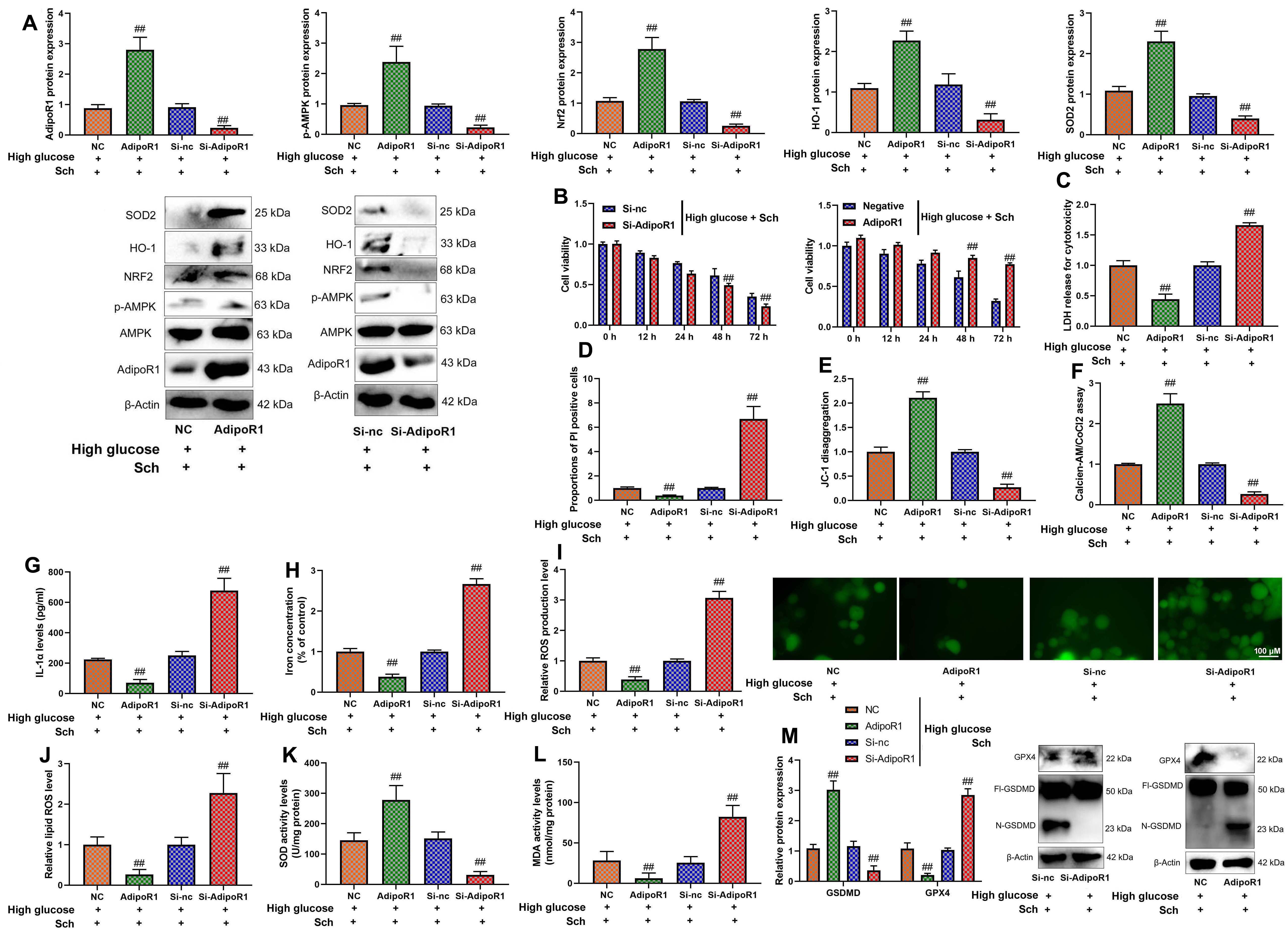

Supplement: Supplementary 3 — Figure S3. Schisandrin A activated AdipoR1/AMPK in vitro model of DN (A) AdipoR1, p-AMPK, Nrf2, HO-1, and SOD2 protein expression in mice model, (B) cell viability, (C) LDH activity level, (D) proportions of PI positive cells, (E) JC-1 disaggregation, (F) calcein-AM/CoCl2 assay, (G) IL-1α levels, (H) iron concentration, (I) ROS protein levels, (J) lipid ROS levels, (K) SOD level, (L) MDA level, and (M) GPX4/GSDMD protein expression in vitro model. Control, sham control mice group; model, in vitro model of DN group; low/med/high, in vitro model of DN by treatment with 25/50/100 μM of Schisandrin A group; Poly, 50 μM of Schisandrin A group; AdipoR1, overexpression of AdipoR1 group; Si-AdipoR1, down-regulation of AdipoR1 group. ##P < 0.01 versus control group; ∗∗P < 0.01 versus in vitro model group; ∗∗∗P < 0.01 versus 50 mg/kg of Schisandrin A group; ###P < 0.01 versus 50 μM of Schisandrin A group. [file 5411462.f3.jpg]

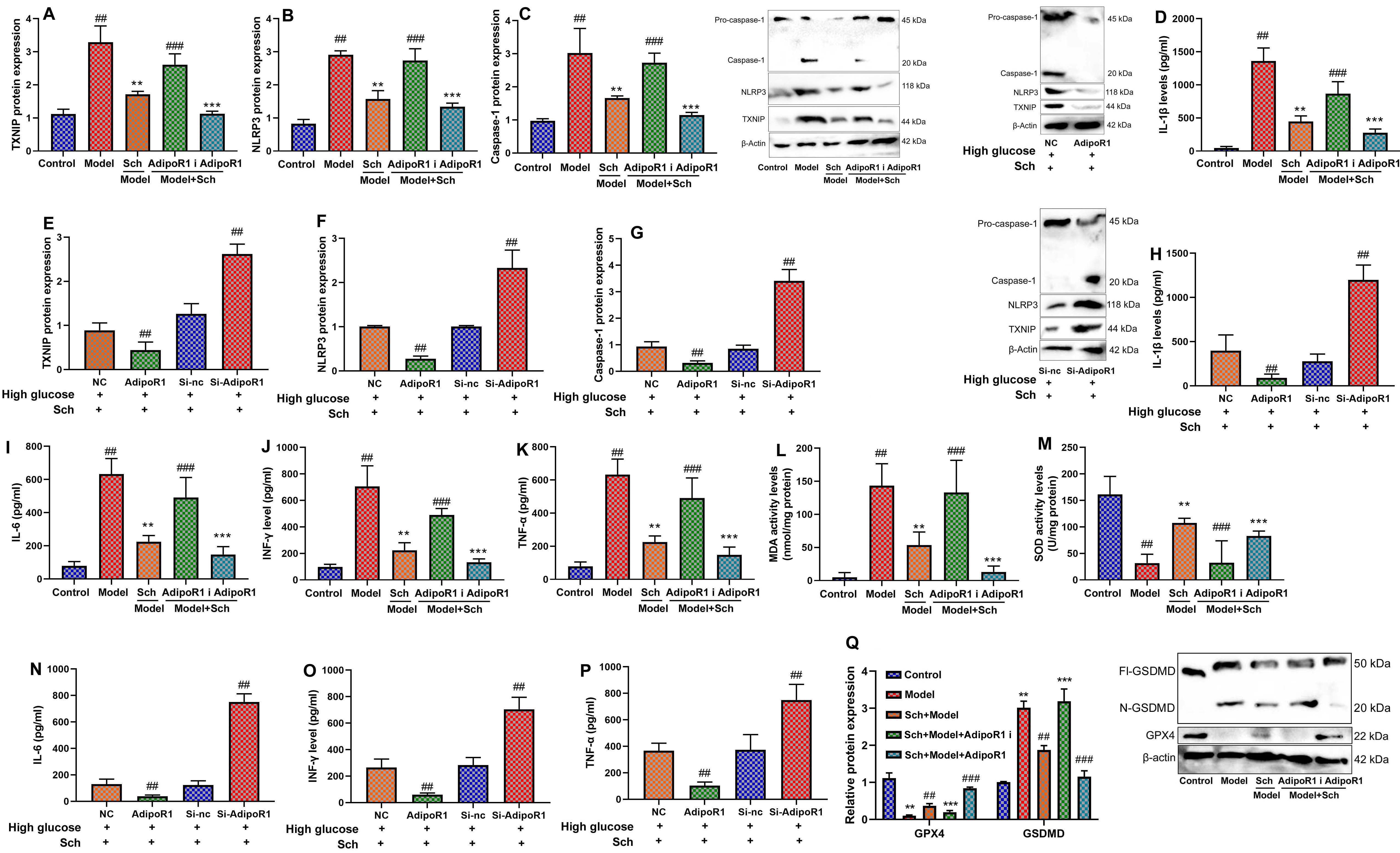

Supplement: Supplementary 4 — Figure S4. The regulation of AdipoR1 affected the effects of Schisandrin A on TXNIP/NLRP3 in model of DN. (A, B, and C) TXNIP, NLRP3, and caspase-1 protein expressions; (D) IL-1β levels in mice model of DN; (E, F, and G) TXNIP, NLRP3 and caspase-1 protein expressions; (H) IL-1β levels in mice model of DN; (I, J, K, L, and M) IL-6, INF-γ, TNF-α, MDA, and SOD levels in mice model of DN; (N, O, and P) IL-6, INF-γ, and TNF-α in vitro model; and (Q) GPX4 and GSDMD protein expression in mice model of DN. Control, sham control mice group; model, STZ-induced mice DN group; low/med/high, mice DN by treatment with 25/50/100 mg/kg of Schisandrin A group; Poly, 50 mg/kg of Schisandrin A group; AdipoR1 i, sh-AdipoR1 group; AdipoR1, AdipoR1 agonist group; ##P < 0.01 versus control group; ∗∗P < 0.01 versus STZ-induced mice DN group; ∗∗∗P < 0.01 versus 50 mg/kg of Schisandrin A group; ###P < 0.01 versus 50 mg/kg of Schisandrin A group. [file 5411462.f4.jpg]
